# Supplementary material for: Enrofloxacin Dose Optimization for the Treatment of Colibacillosis in Broiler Chickens Using a Drinking Behaviour Pharmacokinetic Model
Source: Antibiotics (Basel). 2021 May 19;10(5):604. doi: 10.3390/antibiotics10050604 (PMC8161238; doi:10.3390/antibiotics10050604)
Supplement: Supplementary file 1 [file antibiotics-10-00604-s001.zip › antibiotics-1197556-supplementary.pdf]

SUPPLEMENTARY INFORMATION FILES

S1. Table with secondary PK parameters of enrofloxacin in broilers and their associated standard error (SE), coefficient of variation (CV%) and 95% confidence intervals

Secondary parameters	Estimate	Units	SE	CV%	2.5% CI	97.5% CI
K_e	0.098	1/h	0.004	4.365	0.090	0.106
T_{max}	3.312	h	0.142	4.290	3.033	3.591
$AUC_{(0-inf)}$	21830.100	h*ng/mL	1410.230	6.460	19061.749	24598.451
C_{max}	1546.870	ng/mL	110.586	7.149	1329.784	1763.956
$T_{1/2\ absorption}$	1.012	h	0.076	7.496	0.863	1.161
$T_{1/2\ elimination}$	7.069	h	0.309	4.365	6.464	7.675

Figure S2. Scatterplots of observed concentrations vs. IPRED

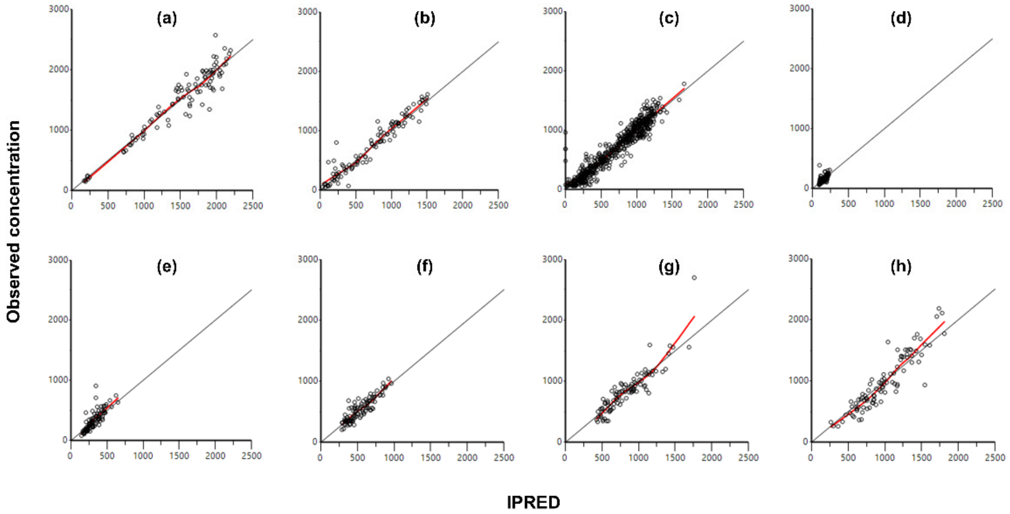

Diagnostic scatterplots of the dependent variable (i.e. the observed concentrations) vs. the individually predicted plasma concentration values (IPRED) on arithmetic scale of the different treatment groups (a = IV, b = PO richly sampled, c = PO sparsely sampled, d = drinking water administration 2.5 mg/kg/24h, e = drinking water administration 5 mg/kg/24h; f = drinking water administration 10 mg/kg/24h, g = drinking water administration 15 mg/kg/24h obtained and h = drinking water administration 20 mg/kg/24h). The black circles represent the observed data, the full black lines are the lines of unity and the red line represents the LOESS line.

Figure S3. Scatterplots of observed concentrations vs. PRED

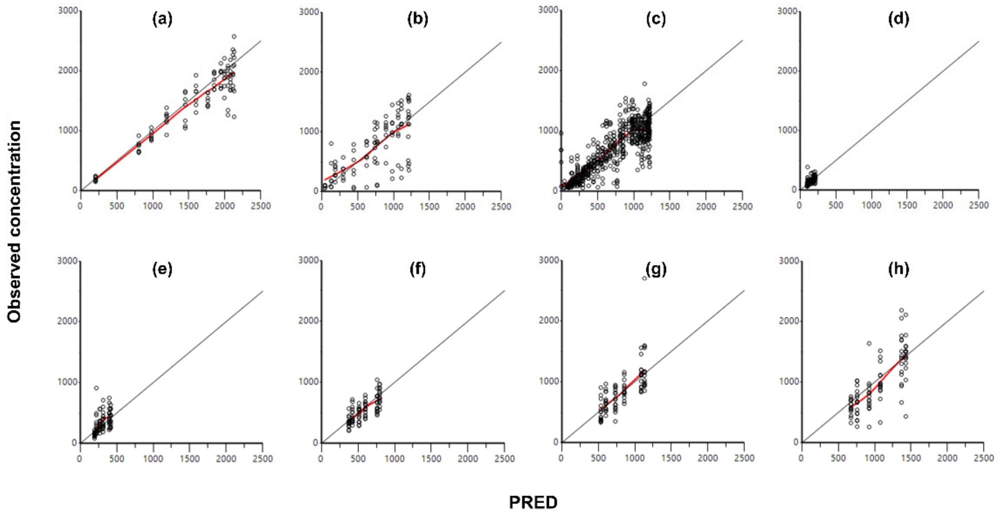

Diagnostic scatterplots of the dependent variable (i.e. the observed concentrations) vs. the predicted plasma concentration values PRED) on arithmetic scale of the different treatment groups (a = IV, b = PO richly sampled, c = PO sparsely sampled, d = drinking water administration 2.5 mg/kg/24h, e = drinking water administration 5 mg/kg/24h; f = drinking water administration 10 mg/kg/24h, g = drinking water administration 15 mg/kg/24h obtained and h = drinking water administration 20 mg/kg/24h). The black circles represent the observed data, the full black lines are the lines of unity and the red line represents the LOESS line.

Figure S4. Scatterplots of CWRES vs. time post administration

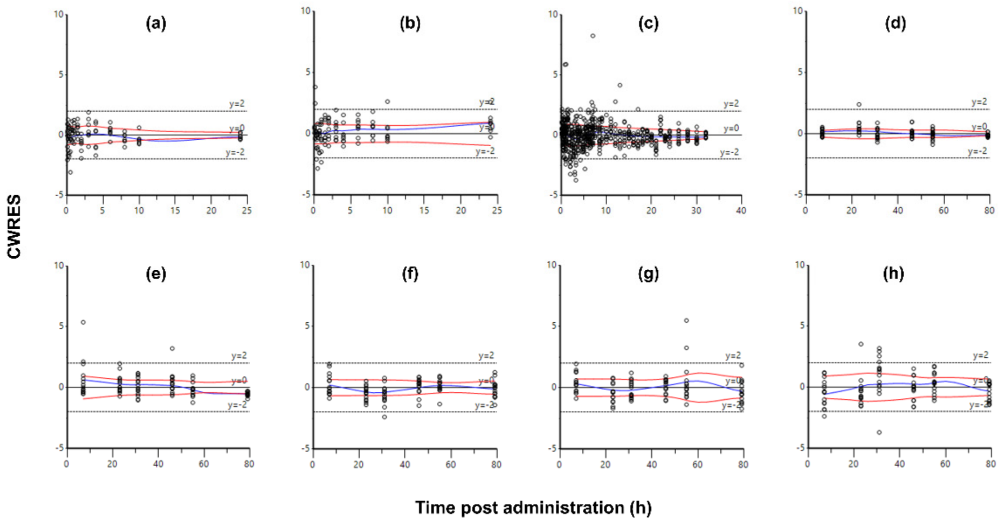

Diagnostic scatterplots of the conditionally weighted residuals (CWRES) vs. time after dose (independent variable, IVAR) of the different treatment groups (a = IV, b = PO richly sampled, c = PO sparsely sampled, d = drinking water administration 2.5 mg/kg/24h, e = drinking water administration 5 mg/kg/24h; f = drinking water administration 10 mg/kg/24h, g = drinking water administration 15 mg/kg/24h obtained and h = drinking water administration 20 mg/kg/24h). Values of CWRES should be concentrated heavily between y = -2 and y = 2. The blue LOESS lines give the overall tendency of the residuals and the red LOESS lines should not show any fanning.

S5. Table of selected quantiles of the PTA analysis of the different doses for a range of MIC values. *The dose where 90% of the population (90% quantile) reached the PK/PD target (fAUC(48-72h)/MIC ≥ 100) for a particular MIC value was considered effective.*

	MIC (µg/mL)	Quantiles (%)				
		25	50	75	90	95
Dose 2.5 mg/kg/24h: AUC(48-72)/MIC	0.032	130.774	116.699	99.793	85.769	76.598
	0.064	66.956	59.750	51.094	43.914	39.218
	0.125	33.478	29.875	25.547	21.957	19.609
	0.25	16.739	14.937	12.773	10.978	9.805
	0.5	8.370	7.469	6.387	5.489	4.902
Dose 5 mg/kg/24h: AUC(48-72)/MIC	0.032	256.270	225.075	196.629	169.099	154.245
	0.064	131.210	115.239	100.674	86.578	78.974
	0.125	65.605	57.619	50.337	43.289	39.487
	0.25	32.803	28.810	25.169	21.645	19.743
	0.5	16.401	14.405	12.584	10.822	9.872
Dose 7.5 mg/kg/24h: AUC(48-72)/MIC	0.032	378.033	333.488	287.445	253.363	229.044
	0.064	193.553	170.746	147.172	129.722	117.270
	0.125	96.776	85.373	73.586	64.861	58.635
	0.25	48.388	42.686	36.793	32.430	29.318
	0.5	24.194	21.343	18.396	16.215	14.659
Dose 10 mg/kg/24h: AUC(48-72)/MIC	0.032	501.455	438.585	365.854	310.930	278.253
	0.064	513.490	449.111	374.635	318.393	284.931
	0.125	128.372	112.278	93.659	79.598	71.233
	0.25	64.186	56.139	46.829	39.799	35.616
	0.5	32.093	28.069	23.415	19.900	17.808
Dose 12.5 mg/kg/24h: AUC(48-72)/MIC	0.032	606.548	521.257	461.387	395.179	353.876
	0.064	310.553	266.884	236.230	202.332	181.185
	0.125	155.276	133.442	118.115	101.166	90.592
	0.25	77.638	66.721	59.057	50.583	45.296
	0.5	38.819	33.360	29.529	25.291	22.648
Dose 15 mg/kg/24h: AUC(48-72)/MIC	0.032	713.504	624.578	545.101	464.987	431.907
	0.064	730.628	639.568	558.184	476.146	442.272
	0.125	182.657	159.892	139.546	119.037	110.568
	0.25	91.328	79.946	69.773	59.518	55.284
	0.5	45.664	39.973	34.886	29.759	27.642
Dose 20 mg/kg/24h: AUC(48-72)/MIC	0.032	917.950	799.749	680.981	574.661	519.756
	0.064	469.991	409.472	348.662	294.226	266.115
	0.125	234.995	204.736	174.331	147.113	133.058
	0.25	117.498	102.368	87.166	73.557	66.529
	0.5	58.749	51.184	43.583	36.778	33.264
Dose 30 mg/kg/24h: AUC(48-72)/MIC	0.032	1260.385	1077.652	886.389	743.419	632.468
	0.064	645.317	551.758	453.831	380.631	323.823
	0.125	322.658	275.879	226.916	190.315	161.912
	0.25	161.329	137.939	113.458	95.158	80.956
	0.5	80.665	68.970	56.729	47.579	40.478
Dose 50 mg/kg/24h: AUC(48-72)/MIC	0.032	1670.091	1358.097	1042.043	799.835	672.603
	0.064	855.087	695.346	533.526	409.515	344.373
	0.125	427.543	347.673	266.763	204.758	172.186
	0.25	213.772	173.836	133.382	102.379	86.093
	0.5	106.886	86.918	66.691	51.189	43.047

S6. Table of the sampling design of the sparsely sampled enrofloxacin PO administration trial

**Experiment 1 (Week 1: 27d of age)**															
	15min	*35min*	*65min*	*1.25h*	*2.5h*	*3h*	*4.25h*	*4.75h*	*5h*	*9h*	*14h*	*15h*	*18h*	*20h*	*24h*
Group 1			X		X				X	X			X		
Group 2		X				X	X				X				X
Group 3	X			X				X				X		X	
**Experiment 2 (Week 1: 29d of age)**															
	10min	*30min*	*45min*	*2h*	*2.75h*	*3.25h*	*4.5h*	*5.25h*	*5.5h*	*8h*	*10h*	*11h*	*19h*	*26h*	*32h*
Group 4		X			X				X	X					X
Group 5			X	X			X					X	X		
Group 6	X					X		X			X			X	
**Experiment 3 (Week 2: 34d of age)**															
	20min	*40min*	*50min*	*1.75h*	*2.25h*	*3.5h*	*4h*	*6.25h*	*6,5h*	*7.5h*	*12h*	*13h*	*17h*	*22h*	*30h*
Group 7			X	X				X				X			X
Group 8		X			X		X				X		X		
Group 9	X					X			X	X				X	
**Experiment 4 (Week 2: 36d of age)**															
	5min	*25min*	*55min*	*1h*	*1.75h*	*3.75h*	*5.75h*	*6h*	*6.75h*	*7h*	*8.5*	*16h*	*21h*	*23h*	*28h*
Group 10			X		X			X			X				X
Group 11		X				X	X			X			X		
Group 12	X			X					X			X		X	
